# Factors Associated With the Accuracy of Depth Gauge Measurements

**DOI:** 10.3389/fsurg.2021.774682

**Published:** 2022-01-13

**Authors:** Pengcheng Liu, Joanna Xi Xiao, Chen Zhao, Xiaodong Li, Guantong Sun, Fei Yang, Xiaoqing Wang

**Affiliations:** ^1^Shanghai Key Laboratory of Orthopedic Implant, Department of Orthopedics, Shanghai Ninth People's Hospital, Shanghai Jiao Tong University School of Medicine, Shanghai, China; ^2^School of Clinical Medicine, The National University of Ireland Galway, Galway, Ireland

**Keywords:** depth gauge, trauma surgery, open reduction and internal fixation, screw length, drilling

## Abstract

**Background:** It is important to select appropriate screws in orthopedic surgeries, as excessively long or too short a screw may results failure of the surgeries. This study explored factors that affect the accuracy of measurements in terms of the experience of the surgeons, passage of drilled holes and different depth gauges.

**Methods:** Holes were drilled into fresh porcine femurs with skin in three passages, straight drilling through the metaphysis, straight drilling through the diaphysis, and angled drilling through the diaphysis. Surgeons with different surgical experiences measured the holes with the same depth gauge and using a vernier caliper as gold standard. The length of selected screws, and the time each surgeon spent were recorded. The measurement accuracy was compared based on the experiences of the surgeons and the passage of drilled holes. Further, parameters of depth gauges and 12-mm cortical bone screws from five different manufacturers were measured.

**Results:** A total of 13 surgeons participated in 585 measurements in this study, and each surgeon completed 45 measurements. For the surgeons in the senior, intermediate, and junior groups, the average time spent in measurements was 689, 833, and 785 s with an accuracy of 57.0, 42.2, and 31.5%, respectively. The accuracy and measurement efficiency were significantly different among the groups of surgeons (*P* < 0.001). The accuracy of measurements was 45.1% for straight metaphyseal drilling, 43.6% for straight diaphyseal drilling, and 33.3% for angled diaphyseal drilling (*P* = 0.036). Parameters of depth gauges and screws varied among different manufacturers.

**Conclusion:** Both observer factor and objective factors could affect the accuracy of depth gauge measurement. Increased surgeon's experience was associated with improvements in the accuracy rate and measurement efficiency of drilled holes based on the depth gauge. The accuracy rate varied with hole passages, being the lowest for angled drilled holes.

## Introduction

Bone drilling and screw selection are usually required in open reduction and internal fixation (ORIF) performed as a surgical treatment for fractures (1, 2). Despite previous studies about optimal drilling parameters to reach maximum accuracy (2–7), there are still concerns about rapid and accurate screw selection intraoperatively. Excessively long screws may rupture the soft tissues and tendons, causing pain and injury to blood vessels and nerves (8–14). It is reported that complications include tendonitis and tendon rupture can occur in 12–23% cases associated with excessively long screws (8). However, if the screw is too short and does not reach the trans cortex, the holding force may be insufficient, resulting in instability of the internal fixation (15). Repeated intraoperative screw replacements due to inaccurate measurements may compromise the threaded trail along the canal, reduce the holding force, possibly leading to a larger threaded “rescue” screw or even failure of the internal fixation (16–19).

Currently, depth gauge, intraoperative fluoroscopy, and preoperative CT are commonly used methods to estimate screw length. The depth gauge is an essential device used in ORIF for anatomically accurate and safe insertion of screws, being widely accepted by orthopedic surgeons. However, few studies have investigated factors that may affect the measurement accuracy of a depth gauge. The most common error is an inaccurate measurement of the length of the drilled hole, subsequently leading to inaccurate screw choice. Using a cadaveric model that mimics clinical situations of ORIF of proximal phalangeal fractures, Jernigan et al. (20) observed that most experienced surgeons were less likely to place short or excessively long screws. Besides, tactile feedback varies due to differences in cortical bone thickness and density at the metaphysis and diaphysis (21) resulting in measurement errors, which makes different passages of drilled holes another factor that influences measurement accuracy. A recent veterinary study also suggested that different depth gauges may affect the accuracy of the measurement (22).

So far, the factors that affect the accuracy of measurement of drilled holes using a depth gauge remains inconclusive. The aim of our study was to further explore factors related to the accuracy of depth gauge measurement. We hypothesized that both observer factor (such as surgeon experience) and objective factors (such as the passage of drilled holes and parameters of the depth gauges) could influence measurement accuracy. To the best of our knowledge, this study is the most comprehensive one concerning factors related to the accuracy of depth gauge measurement. Moreover, we introduce the indicator of efficiency of measurements for the first time, taking into account both the speed and accuracy rate of measurements.

## Materials and Methods

### Study Design

To simulate real surgical operations, the dorsal part of three fresh porcine femurs with skin was subjected to a straight longitudinal skin incision. Soft tissues on the lateral aspect were separated, exposing the diaphysis and metaphysis of the femur. Holes were drilled latero-medially along the length of a bone from proximal femur to distal end using a 3.2 mm diameter drill bit. For each bone, holes were drilled with three different passage types in random order, namely straight drilling through the metaphysis, straight drilling through the diaphysis, and angled drilling through the diaphysis. Finally, all holes broke through the trans cortex and 45 holes were used, with 15 holes in each type of passage. 7 holes were excluded because 6 were too close to their adjacent holes, and 1 perforated the joint.

A total of 13 orthopedic trauma surgeons participated in the experiment and were divided into three groups according to their experience or training level: senior group (3 surgeons with ≥10 years after medical school graduation), intermediate group (4 surgeons with 5–10 years after medical school graduation), and junior group (6 surgeons within 5 years of medical school graduation). Each surgeon was asked to use the same depth gauge with a 110-mm-long scale (319.100, Synthes) on each drilled hole, then report the screw length for the respective hole, with options consisting of integral increments of 1 mm (and without consideration of plate variables). In parallel, the total time each surgeon took to measure all 45 holes was recorded.

### Definition of Ideal Screw Length

**Figure 1:**
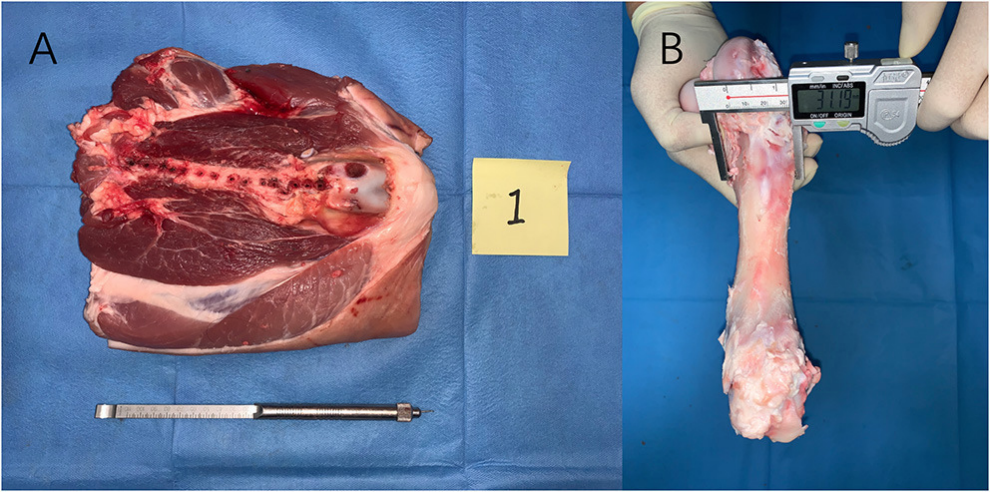
**(A)** To simulate surgical operations, a dorsal part of three fresh porcine femurs with skin was accessed using a straight longitudinal skin incision. **(B)** After all measurements were finished the soft tissue was peeled off, and the holes were measured with a calibrated vernier caliper with 0.01 mm precision and scale from 0 to 150 mm (0122032, 355-101, Hengliang, Shanghai, China); the result of which was defined as the actual length of the hole.

**Figure 2:**
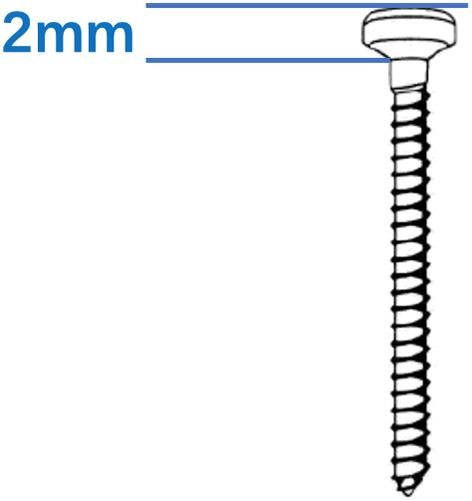
The threaded portion of the screw was equal to the length of the screw without correction for head height, which was 2 mm in this study.

To determine whether the selected screw length was ideal, porcine femurs were stripped of all soft tissue, and then measured by two observers (XQW and PCL) using a calibrated vernier caliper with 0.01 mm precision and scale from 0 to 150 mm (0122032,355-101, Hengliang, Shanghai, China), the average of which was used as the actual measurement of the hole (Figure 1). In theory, an ideal screw is the smallest available size in which screw threads perforate and sustain the trans cortex. We assumed the threaded portion of the screw was equal to the length of the screw without correction for head height, which was 2 mm in this study. Comparing the results of the depth gauge and the vernier caliper, the position of the screw tip relative to the trans cortex was determined, that is, the length of the screw tip to the trans cortex = (length of the selected screw−2 mm)—the actual measured value of the hole (Figure 2). Ideal screws were those in which the tip of the screw reached the surface of the trans cortex but did not protrude more than 1.0 mm beyond it, with a positive length of the screw tip on the trans cortex. Short screws were those that failed to reach the trans cortical surface, with a negative length of the screw tip on the trans cortex.

Theoretically, sufficient holding force can be obtained when the screw reaches the ideal length of the screw. Prior biomechanical data have demonstrated that screws that extend past the volar cortical surface have higher pull-out strength than screws that do not extend past the surface ^18^ and that there was no marginal increase in pull-out strength for screws that extended beyond 1 mm past the volar surface of the bone.

The accuracy of measurements was determined as the number of screws with an ideal length divided by the total number of measurements.

The efficiency of measurements was determined as the number of screws with an ideal length divided by the total time spent by each surgeon (unit: number of screws per minute).

### Measurement of Different Depth Gauges and Screws

**Figure 3:**
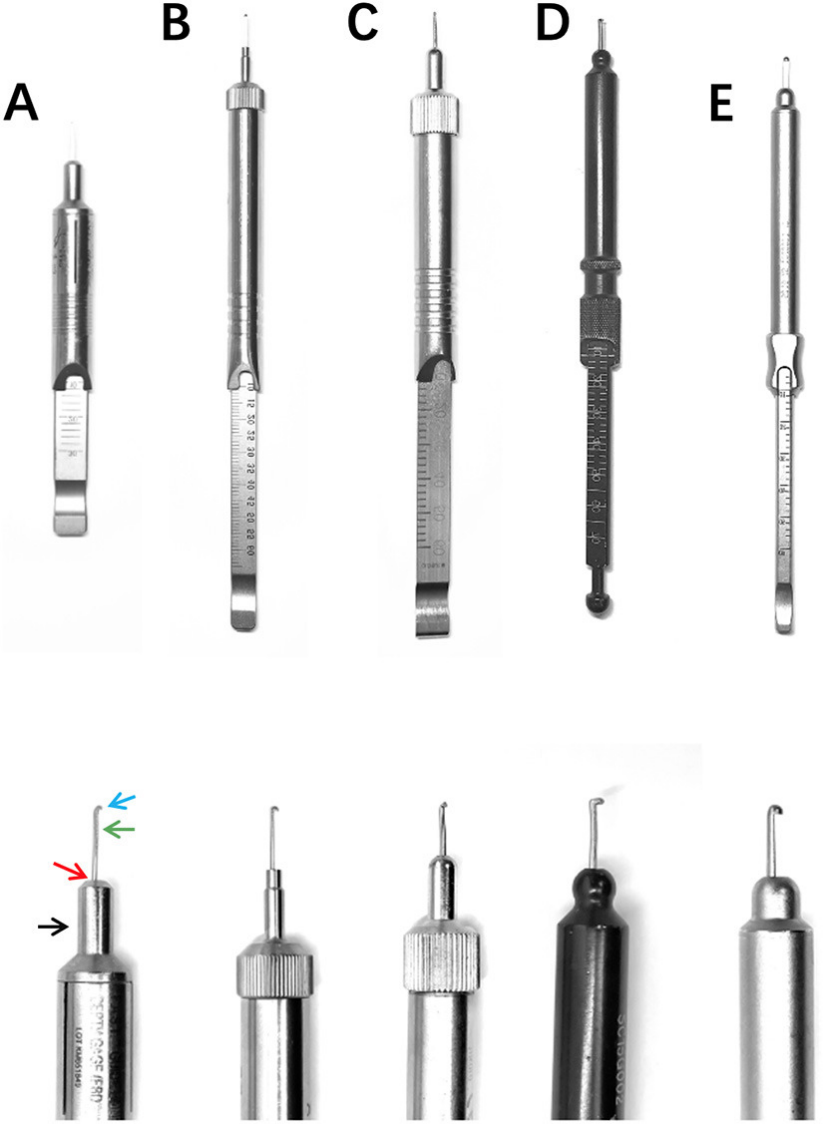
Depth gauges used in our study. **(A)**: KM651849, DOUBLE MEDICAL; 5.70-mm-diameter base shaft (black arrow), 45° bevel (red arrow), 4.00-mm-diameter base end, 0.82-mm-width rod on the lateral view (green arrow) and 1.30-mm-width hook (blue arrow). **(B)**: 191003255, WEGO; 2.46-mm-diameter base shaft (yellow arrow) with a flat base end, 0.58-mm-width rod on the lateral view and 1.37-mm-width hook. **(C)**: 319.010, Synthes; 5.45-mm-diameter base shaft, rounded bevel, 1.78-mm-diameter base end, 1.01-mm-width rod on the lateral view and 1.32-mm-width hook. **(D)**: SC15G002, Zimmer Biomet; 6.30-mm-diameter base shaft, rounded bevel, 1.21-mm-diameter base end, 0.99-mm-width rod on the lateral view and 2.18-mm-width hook. **(E)**: SY 9125, Shuangyang, Jiangsu, China; 5.98-mm-diameter base shaft, rounded bevel, 1.19-mm-diameter base end, 0.93-mm-width rod on the lateral view and 1.85-mm-width hook.

**Figure 4:**
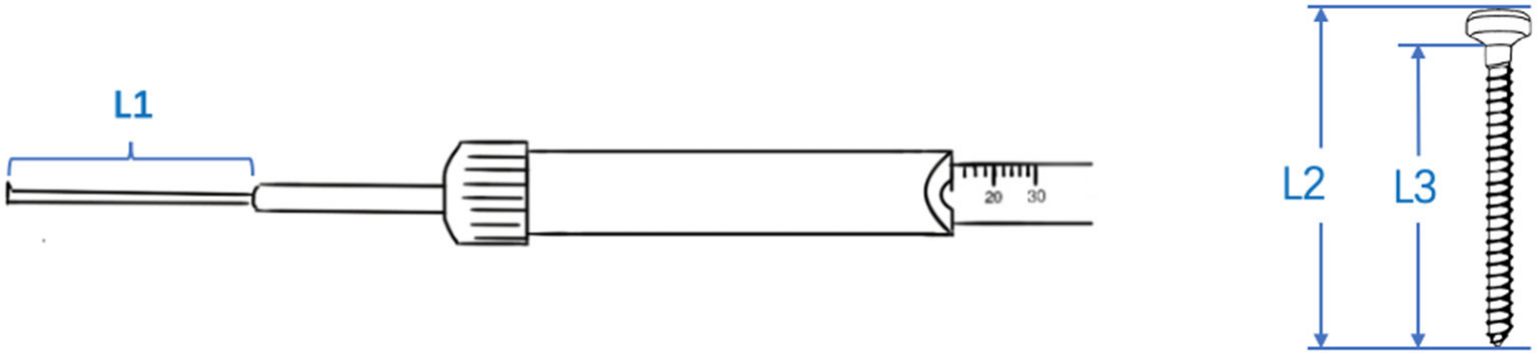
Depth gauges from five manufacturers were placed on a 12 mm scale to measure the length of the rod (L1) and the length of 12 mm cortical bone screws [including its full length (L2) and length of the body part (L3)].

To evaluate the influence of different depth gauges on measurement accuracy, six depth gauges from four different manufacturer (319.010, Synthes; SY 9125, Shuangyang, Jiangsu, China; SC15G002, Zimmer Biomet; KM651849, DOUBLE MEDICAL; 191003255, WEGO) (Figure 3) and five 12-mm cortical bone screws (404.812, Synthes; 0106936794823478, Shuangyang; 815037012, Zimmer Biomet; 040010022, DOUBLE MEDICAL; 6133512, WEGO) were collected for further study. Each depth gauge was set to read 12 mm on it's scale bar. The length of the rod (L1) and the length of the 12-mm cortical bone screws [including its full length (L2) and length of the body part (L3)] (Figure 4) were measured by one observer (XQW) using a calibrated vernier caliper with 0.01 mm precision and scale from 0 to 150 mm (01130048,349-055, Hengliang, Shanghai, China). L1, L2, and L3 were compared within the same system.

### Statistical Analysis

Chi-square tests were used to analyze differences in the accuracy of measurements between surgeons and passages. One-way ANOVA was used to compare the efficiency of measurements among the groups of surgeons. *P* < 0.05 was considered statistically significant.

## Results

### Accuracy and Efficiency of Measurements Between Surgeons

**Figure 5:**
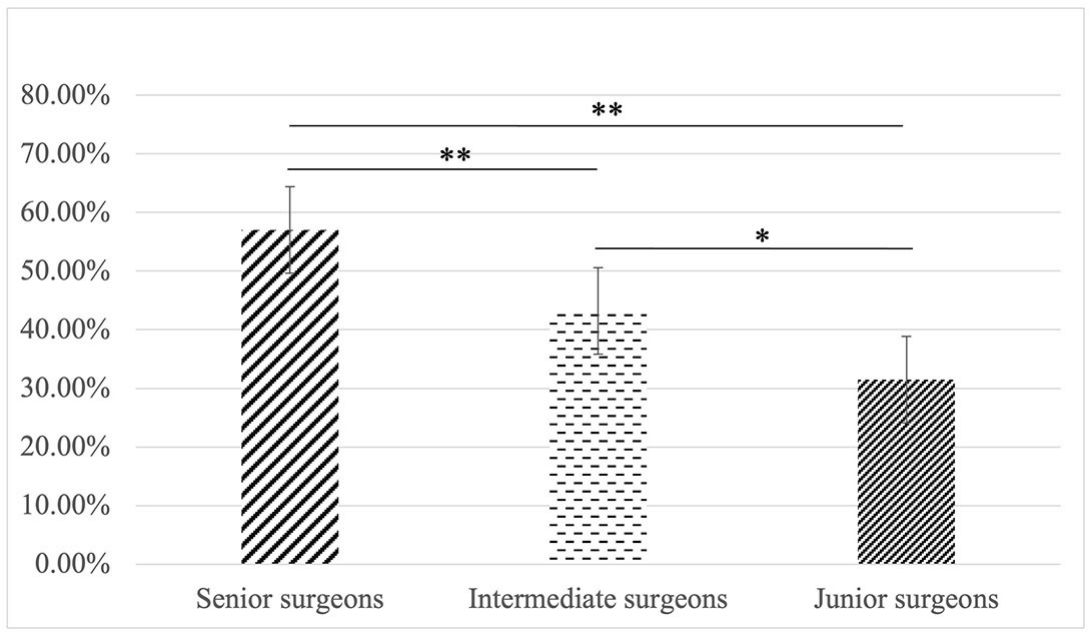
The most experienced surgeons demonstrated a higher accuracy than those in the intermediate (77/135, 57% vs. 76/180, 42.2%, *P* = 0.009) and junior (77/135, 57% vs. 85/270, 31.5%, *P* < 0.001) group. Likewise, surgeons in the intermediate group had a higher accuracy than that of the junior group (76/180, 42.2% vs. 85/270, 31.5%, *P* = 0.020), error bar indicate 95% confidence intervals. *p < 0.05, **p < 0.01.

A total of 13 surgeons participated in 585 measurements and each surgeon completed 45 measurements (Figure 5). The ideal screws were defined as a screw that reached the trans cortex but did not protrude more than 1 mm beyond it. Three senior surgeons completed 135 measurements, taking on average 689 s for 45 measurements with 77 ideal screws placed and an accuracy rate of 57.0%. Four intermediate surgeons completed 180 measurements, taking on average 833 s for 45 measurements with 76 ideal length screws placed and an accuracy rate of 42.2%. Finally, 6 junior surgeons completed 270 measurements, taking on average 785 s for 45 measurements with 85 ideal screws placed and an accuracy rate of 31.5%.

The accuracy of measurements varied with the experience of surgeons (*p* < 0.001) (Table 1), in which the most experienced surgeons demonstrated a higher accuracy than those in the intermediate (77/135, 57% vs. 76/180, 42.2%, *P* = 0.009) and junior (77/135, 57% vs. 85/270, 31.5%, *P* < 0.001) group. Likewise, surgeons in the intermediate group had a higher accuracy than that of the junior group (76/180, 42.2% vs. 85/270, 31.5%, *P* = 0.020). Therefore, with more experience, the accuracy rate and efficiency of measurements using the depth gauge significantly improved (*P* = 0.033).

**Table 1:** Accuracy and efficiency of measurements between surgeons.

	**Senior**	**Intermediate**	**Junior**	***P*-value**
Number of accurate measurements	77	76	85	
Accuracy rate	77/135 (57.0%)	76/180 (42.2%)	85/270 (31.5%)	*P* < 0.001
Number of accurate measurements (seconds)	689	833	785	
Efficiency (number of screws per minute)	2.28	1.46	1.26	*P* = 0.033

*The accuracy and efficiency of measurements varied with the experience of surgeons (p < 0.001). Increased surgeon's experience was associated with improvements in the accuracy rate and measurement efficiency of drilled holes*.

### Relationship Between the Accuracy Rate of Measurements and the Location and Passages of Drilled Holes

**Figure 6:**
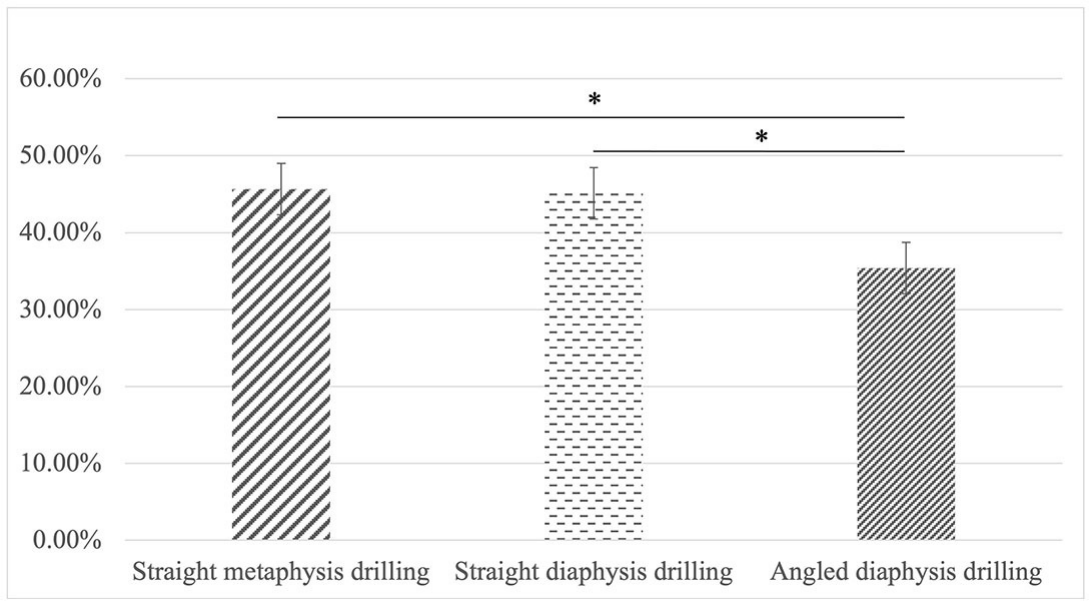
There was no statistical difference between straight metaphyseal and diaphyseal drilling (85/195, 43.6% vs. 88/195, 45.1%; *P* = 0.760). Meanwhile, the accuracy of measurements was significantly lower for angled diaphyseal drilling when compared to that of straight diaphyseal drilling (65/195, 33.3% vs. 85/195, 43.6%; *P* = 0.037) and straight metaphyseal drilling (65/195, 33.3% vs. 88/195, 45.1%; *P* = 0.017). *p < 0.05.

Of the 585 measurements, 195 were made with straight drilling through the metaphysis, for which 88 screws were at an ideal length (accuracy rate of 45.1%). For straight drilling through the diaphysis 85 screws were at an ideal length (accuracy rate of 43.6%), whereas for angled drilling through the diaphysis, 65 screws were at an ideal length (accuracy rate of 33.3%). Interestingly, the accuracy rate of measurements varied with the location and passage of the drilled holes (*P* = 0.036) but there was no statistical difference between straight metaphyseal and diaphyseal drilling (85/195, 43.6% vs. 88/195, 45.1%; *P* = 0.760). Meanwhile, the accuracy of measurements was significantly lower for angled diaphyseal drilling when compared to that of straight diaphyseal drilling (65/195, 33.3% vs. 85/195, 43.6%; *P* = 0.037) and straight metaphyseal drilling (65/195, 33.3% vs. 88/195, 45.1%; *P* = 0.017) (Figure 6).

### Measurement of Different Depth Gauges and Screws

The results showed that L1 values were 10.30 mm (319.010, Synthes), 9.05 mm (Shuangyang, Jiangsu, China), 10.05 mm (SC15G002, Zimmer Biomet), 12.03 mm (KM651849, DOUBLE MEDICAL) and 9.30 mm (191003255, WEGO). The L2 values were 12.30 mm (404.812, Synthes), 12.00 mm (0106936794823478, Shuangyang), 13.50 mm (815037012, Zimmer Biomet), 12.00 mm (040010022, DOUBLE MEDICAL) and 11.98 mm (6133512, WEGO). The L3 values were 10.30 mm (404.812, Synthes), 10.40 mm (0106936794823478, Shuangyang), 11.10 mm (815037012, Zimmer Biomet), 10.45 mm (040010022, DOUBLE MEDICAL) and 9.62 mm (6133512, WEGO) (Table 2).

**Table 2:** Matching degree between depth gauge and screws from different manufacturers.

**Manufacturer**	**Length of the rod (L1, mm)**	**Full length of the screw (L2, mm)**	**Length of the body part (L3, mm)**
Synthes	10.30	12.30	10.30
Shuangyang	9.05	12.00	10.40
Zimmer biomet	10.05	13.50	11.10
Double medical	12.03	12.00	10.45
WEGO	9.30	11.98	9.62

*Depth gauges from five manufacturers were placed on a 12 mm scale to measure the length of the rod (L1) and the length of 12 mm cortical bone screws [including its full length (L2) and length of the body part (L3)]*.

## Discussion

This study explored both observer factor and objective factors (the passages of the drilled holes, depth gauge from different manufacturers) associated with measurement accuracy with vernier calipers used as a gold standard.

There were statistical differences in the accuracy rate of measurements among different groups of surgeons, with more experienced surgeons having more accurate measurements. However, there was no significant difference in the time taken for each surgeon to measure 45 holes.

To further compare surgeons' skills, we calculated the efficiency of measurements (i.e., the number of holes accurately measured using the depth gauge per minute). Considering both the speed and accuracy rate of measurements, our results showed that the measurement efficiency was positively correlated with surgeon training, indicating that theoretical study and simulated operations of a depth gauge may be helpful to improve the accuracy of measurements among the surgeons. Other studies have also shown that it was helpful for residents to master their surgical skills in advance either in the form of lectures or simulated operations (23–30).

Using a cadaveric model that simulates ORIF of proximal phalangeal fractures, Jernigan et al. (20) measured drilled holes using a depth gauge without fluoroscopy assistance and studied the relationship between the level of training and the rates of ideal screw length selection among the surgeons. Ideal screws were defined as a screw that reached the volar cortex but did not protrude more than 1 mm beyond it and the study results showed that for 18 participants and a total of 648 selected screws, there was no relationship between the rate of ideal screw selection and level of training. Attending surgeons were less likely to place short screws or screws protruding more than 1 mm beyond the volar cortex. These results are in contrast to our analysis possibly because they used the proximal phalanx that has a short average transverse diameter and thin soft tissue on the opposite side, thus tactile feedback would be more evident. Also, the surgeons were more familiar with the anatomy of the proximal phalanx, which may also affect the accuracy of measurements. We used porcine femurs with skin, which have a relatively large average transverse diameter and thicker soft tissue, and the surgeons were relatively unfamiliar with the anatomy, thus the accuracy of the measurements was only related to the skill of the surgeons. Taken together, the accuracy rate of measurements may vary for different bones when selecting an ideal screw using the depth gauge, hence, this needs to be explored in future studies.

Our study also found no statistical difference in the accuracy rate of measurements between straight holes drilled through the diaphysis or metaphysis. However, the accuracy rate was significantly lower for angled drillings through the diaphysis, which may be attributed to a different situation in the measurement when the tip of the depth gauge hooked to the obtuse or acute angle of the fracture. According to the trauma treatment principle of the Association for the Study of Internal Fixation (AO/ASIF), the tip of the depth gauge should be hooked to the obtuse angle of the fracture in angled drilling holes, which, unfortunately, is often ignored by surgeons during the operation. Demsey et al. (21) drilled holes in three different clinically relevant conditions: straight drilling through the diaphysis, angled drilling through the diaphysis, and straight drilling through the metaphysis using laser range-finding sensors in pig bones and laser range-finder-based prototypes for depth measurements. The results showed that the accuracy of the device was lowest for straight diaphyseal drilling but the same for angled diaphyseal and straight metaphyseal drillings, which is contrary to our findings. This discrepancy may have occurred because the prototype device is still in its experimental stage, so the accuracy and algorithm may not be optimal. Also, laser displacement sensors were mounted on the surgical drill on either side of the drilling access in the horizontal plane and the laser gauge provided continuous displacement measurements relative to the surface illuminated by the laser line. The difference in distance estimates between the breach of the second cortex and the initial position was taken as the bore depth, therefore the accuracy differed in measuring the depth of drilled holes in bones compared with a conventional depth gauge.

Interestingly, depth gauges produced by different manufacturers yielded contrasting measuring values according to our measurements. The portion of the valid length, which is the body part of the screw, varied among different manufacturers and the actual length of the measurements using depth gauges were not strictly fit with the length of the ‘selected screws’ even within the same system. Moreover, the parameters of the depth gauges in our study varied with each other. McChesney et al. (22) mentioned that the amount of recess of the depth gauge base within a plate hole is influenced by the size and geometry of the depth gauge base and plate hole. A depth gauge with small-diameter base or long bevel may cause underestimate of the measurement. Further study could further investigate the influence of the geometry and width of the hook on the accuracy of measurement. A wide hook seems to have better feedback when measuring. Thus, surgeons should improve their understanding of the parameters of different manufacturers, systems, and screws in clinical practice to help in the selection of an ideal length screw based on a depth gauge.

**Figure 7:**
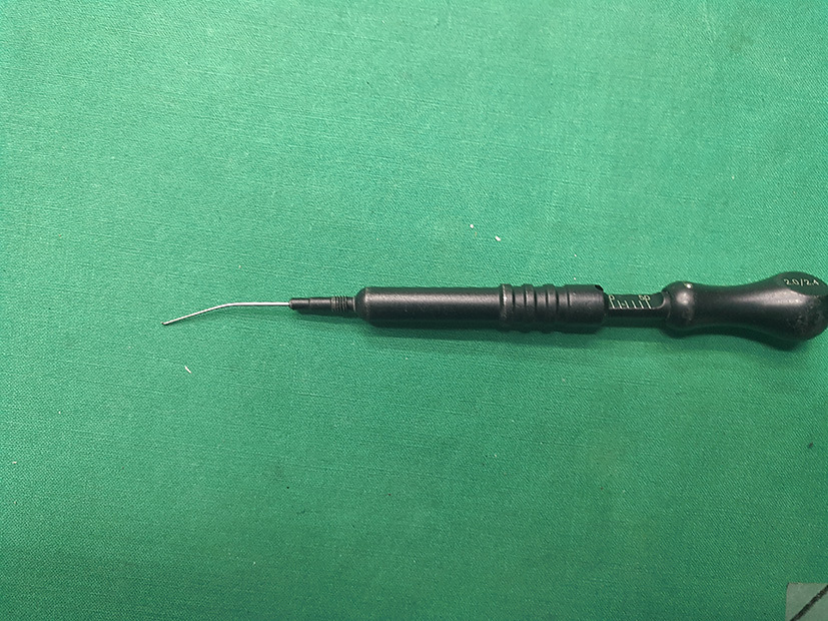
The front probes of depth gauges may bend due to improper use, which may also lead to inaccurate measurements.

The working state of the depth gauge may also affect the accuracy of the measurements. Rafique et al. (31) reported that an incorrect assembly of the depth gauge metal collar led to measurement errors. Also, the front probes of some depth gauges may bend due to improper use (Figure 7), which may also lead to inaccurate measurements. Therefore, before using the depth gauge, surgeons must check its state to avoid similar mechanical failures affecting the measurement accuracy.

Although our study explored effects related to the accuracy of measurements using a depth gauge, there were still some limitations. First, porcine femurs with skin rather than cadaver bone were used in this study, and their tactile feedback may be different. Porcine femurs are often used in teaching operations in orthopedics and are regarded as having similar components to the human bone (21, 26). In our study, we used porcine femurs with skin and kept the surrounding soft tissue to simulate a real surgical scene. Moreover, the screws were not truly screwed into the bones. Instead, the ideal length of the screw was determined only by the measurement using the depth gauge. However, in clinical practice, the surgeon can be assisted by the torsion force when the screw breaks through the trans cortex of the bone. It is crucial to determine whether the screw length is suitable when using the depth gauge for the first time as screw replacement may loosen the canal and decrease the screw grip. For an individual patient, the density of the bone, the type, and site of the fracture, as well as the diameter of the screw may all affect the holding force of the screw. According to Battula et al. (10), in normal bones, the depth of insertion of the tip of the screw should be ~1 mm past the far cortex, while in osteoporotic bone, this should be at least 2 mm past the far cortex. However, Schoenfeld et al. (32) found that the biomechanical conclusions of self-tapping screws simulated on artificial or corpse bone could not be applied to clinical practice, and for people with either a healthy bone or osteoporosis, the depth of insertion of the tip of the screw for adequate fracture fixation was at least 2 mm past the far cortex. In our study, the holding force and the protection of soft tissue were considered, therefore, the ideal screw was defined as a screw that reached the trans cortex but did not protrude more than 1 mm, with a certain guiding significance in clinical practice.

## Conclusion

Screw selection with appropriate length is crucial in orthopedic surgery. Based on our findings, both observer factor and objective factors could affect the accuracy of depth gauge measurement. Increased surgeon's experience was associated with improvements in the accuracy rate and measurement efficiency of drilled holes based on the depth gauge. Theoretical study and simulated operations of a depth gauge may be helpful to improve the accuracy of measurements among the surgeons. The accuracy rate varied with hole passages, being the lowest for angled drilled holes. Moreover, Parameters of depth gauges and screws varied among different manufacturers.

## Data Availability Statement

The raw data supporting the conclusions of this article will be made available by the authors, without undue reservation.

## Ethics Statement

Ethical review and approval was not required for the animal study as purchased porcine femurs were used, which does not involve living animals.

## Author Contributions

PL, JX, and XW: conception and design of the research. PL, JX, and CZ: writing of the manuscript. PL, CZ, and FY: acquisition of data. PL, CZ, XL, and GS: analysis and interpretation of the data and statistical analysis. XW: obtaining financing and critical revision of the manuscript for intellectual content. All authors contributed to the article and approved the submitted version.

## Funding

This work was supported by the National Natural Science Foundation of China (grant numbers 81871791 and 81272036).

## Conflict of Interest

The authors declare that the research was conducted in the absence of any commercial or financial relationships that could be construed as a potential conflict of interest.

## Publisher's Note

All claims expressed in this article are solely those of the authors and do not necessarily represent those of their affiliated organizations, or those of the publisher, the editors and the reviewers. Any product that may be evaluated in this article, or claim that may be made by its manufacturer, is not guaranteed or endorsed by the publisher.
